# Higher monocyte count with normal white blood cell count is positively associated with 10-year cardiovascular disease risk determined by Framingham risk score among community-dwelling Korean individuals

**DOI:** 10.1097/MD.0000000000015340

**Published:** 2019-04-26

**Authors:** Jung-Hwan Kim, Yong-Jae Lee, Byoungjin Park

**Affiliations:** a Department of Family Medicine, Yonsei University College of Medicine, 50–1 Yonsei-ro, Seodaemoon-gu; b Department of Family Medicine, Gangnam Severance Hospital, 211 Eonju-ro, Gangnam-gu, Seoul; c Department of Family Medicine, Yongin Severance Hospital, 225 Gumhak-ro, Cheoin-gu, Yongin, Gyeonggi-do, Republic of Korea.

**Keywords:** cardiovascular disease, Framingham risk score, Korean, monocyte counts

## Abstract

The cardiovascular disease (CVD) has been identified as a leading cause of premature mortality among middle-aged and elderly individuals globally. Inflammation plays an important role in aging and age-related diseases, such as atherosclerosis and CVD. White blood cell (WBC) count is an inexpensive, simple biomarker of systemic inflammations and includes several cell subtype counts, such as neutrophils, monocytes, lymphocytes, basophils, and eosinophils. However, which component of a WBC count has the ability to predict CVD remains controversial. The objective of this study was to assess the association between monocyte counts and 10 year-CVD risk among community-dwelling Korean individuals using the Framingham risk score (FRS). We studied a total of 627 participants aged over 30 years who underwent routine health examinations. The mean age of the study population was 48.1 ± 11.7 years, and 56.9% were male. In the multiple regression analysis, the independent contribution of monocyte count to Framingham 10-year CVD risk was 0.217 ± 0.092 (*P* = .018) after adjusting for confounding variables. We found that of the various WBCs, monocyte count is an independent predictor of CVD risk. Further larger-scale prospective cohort studies are warranted to determine these associations in the future.

## 1 Introduction

Inflammation is an integrated reaction and a major defensive mechanism against the disturbance of homeostasis in infectious and injurious conditions.^[1]^ Recent studies have shown that inflammation plays an important role in disabilities, cancer, aging, and age-related diseases, such as atherosclerosis and cardiovascular disease.^[2]^

Cardiovascular disease (CVD) is a major health problem and the leading cause of death around the world.^[3]^ It is estimated that CVD will increase by 10% in the next 20 years.^[4]^ Therefore, it is important to assess preventive measures to minimize CVD risk. Several markers of inflammation have been studied as predictors of CVD and mortality.^[5]^

A comprehensive white blood cell (WBC) count includes 5 subtypes: neutrophils, monocytes, lymphocytes, basophils, and eosinophils. All WBCs play various roles in the inflammatory response and host immunity.^[6]^ Increased total WBC count is positively associated with CVD, stroke and all-cause mortality.^[7,8]^ However, it remains unclear which component of the WBC count has the ability to predict CVD. The main cause of CVD is atherosclerosis, which is defined as the chronic formation of plaques in blood vessels.^[9]^ Monocytes are a part of the mononuclear phagocytic system, with roles in development, inflammation, and host immunity.^[10]^

The Framingham risk score (FRS) is a simple and feasible method used for prediction of CVD severity.^[11]^ The objective of this study was to assess the relationship between monocyte counts and 10-year CVD risk among community-dwelling Korean individuals using the FRS.

## 2 Methods

### 2.1 Study population

We retrospectively reviewed the medical records of 641 sequential subjects older than 30 years of age who voluntarily received a health examination at the Health Promotion Center at Gangnam Severance Hospital, Seoul, Korea from January 2007 through November 2008. Subjects aged 30 years or older were included, as the FRS can be calculated for this population.^[12]^ Subjects meeting any of the following criteria were excluded (n = 14): subjects with white blood cell counts of less than 3.0 or more than 1.1 × 10^3^ cell/μL; subjects with C-reactive protein levels of more than 10.0 mg/L; and subjects with any acute inflammatory disease. Finally, 627 subjects (357 men, 270 women) were included in our analysis. This study was approved by the Institutional Review Board of Yonsei University College of Medicine, Gangnam Severance Hospital (IRB No: 3–2018–0266). All subjects provided informed consent.

### 2.2 Data collection

The examinations were performed by medical staff according to standard procedures. Participants were asked about lifestyle behaviors, including cigarette smoking, alcohol consumption, and physical activity. They were also questioned about whether they were currently undergoing treatments for any disease and, if so, they were asked for the date of diagnosis and a list of current medications. Trained staff reviewed the completed questionnaires and entered the responses into a database. Participants were categorized as non-smokers, ex-smokers, or current smokers. Subjects were also classified as non-drinkers/abstainers (<140 g/week) or current drinkers (≥140 g/week). Height and body weight were measured to the nearest 0.1 cm and 0.1 kg in light indoor clothing and without shoes. Body mass index (BMI) was calculated as the ratio of weight (kg)/height (m^2^). Blood pressure was measured with the participant in a sitting position after 5 minutes of rest using an automated device (TM-2665P, A&D Co., LTD., Tokyo, Japan). Hypertension was defined as a history of taking hypertension medication, systolic blood pressure ≥140 mmHg, or diastolic blood pressure ≥90 mmHg. Diabetes was defined as a history of taking diabetes medication or a fasting plasma glucose level ≥126 mg/dL. After a 12-hour overnight fast, blood samples were taken from an antecubital vein. Tubes containing EDTA were used for the whole blood count. WBCs were quantified by an automated blood cell counter (ADVIA 120, Bayer, NY) within 1 hour of blood sampling. Fasting plasma glucose, total cholesterol, triglycerides, high-density lipoprotein (HDL) cholesterol, aspartate aminotransferase, alanine aminotransferase, and uric acid levels were measured using a Hitachi 7600–110 Chemistry system Autoanalyzer (Hitachi, Tokyo, Japan).

The modified National Cholesterol Education Program Adult Treatment Panel III (NCEP-ATP III) was used to define metabolic syndrome. Because waist circumference was not measured, we defined obesity as BMI ≥ 25 kg/m^2^, as suggested by the position statement of the American College of Endocrinology.^[13]^ Therefore, metabolic syndrome was defined by the presence of three or more of the following risk factors: obesity with BMI ≥25.0 kg/m^2^; elevated systolic blood pressure ≥130 mmHg, elevated diastolic blood pressure ≥85 mmHg, or use of an anti-hypertensive medication; high fasting plasma glucose ≥100 mg/dL or use of an anti-diabetes medication; high triglycerides ≥150 mg/dL; and low HDL cholesterol <40 mg/dL for men and <50 mg/dL for women.

### 2.3 Definition of the Framingham risk score (FRS)

The FRS, a widely-accepted tool, is derived from the Framingham Heart Study and used to predict the risk of coronary disease, such as angina, myocardial infarction, cardiovascular death over a 10-year period. This risk assessment is a sex-specific method determined with the following set of variables: sex (male/female), age (years), systolic blood pressure (mmHg), treatment for hypertension (yes/no), diabetes status (yes/no), current smoker (yes/no), total cholesterol (mg/dL), and HDL cholesterol (mg/dL).^[14]^ The FRS is composed of both modifiable (smoking, systolic blood pressure, use of antihypertensive medication, dyslipidemia, diabetes status) and not-modifiable (age, sex) risk factors. Individuals’ scores, representative of their 10-year risk of developing CVD and calculated based on their total points, are typically categorized as low (FRS < 10%), intermediate (FRS = 10–20%), or high (FRS > 20%).^[15]^

The FRS is a consistent, precise risk score that has been validated in large population studies.^[16]^ In addition, recent studies have mentioned that the FRS is a reasonable alternative for use in multiethnic groups.^[17]^ This risk score can be useful for both patient education and clinicians deciding whether lifestyle modification or medical treatment is warranted.^[18]^ The FRS was calculated using a computer program.^[19]^

### 2.4 Statistical analysis

All analyses were conducted using SAS statistical software, version 9.4 (SAS Institute Inc, Cary, NC). The demographic and biochemical characteristics of the study population were analyzed using independent 2-sample *t* tests for continuous variables and the chi-squared tests for categorical variables. A multiple regression analysis was performed in order to determine whether monocyte count is an independent determinant for an increased Framingham 10-year CVD risk after adjusting for other confounding variables. All statistical tests were 2-sided, and statistical significance was determined at *P* < .05.

## 3 Results

Table 1 shows the clinical and chemical characteristics of the 357 men and 270 women. The mean age of the study population was 48.1 ± 11.7 years, and the mean BMI was 23.8 ± 2.9 kg/m^2^. Systolic blood pressure, diastolic blood pressure, fasting plasma glucose and triglyceride levels, Framingham 10-year CVD risk, WBC count and monocyte count were all higher in men compared to women.

**Table 1:**
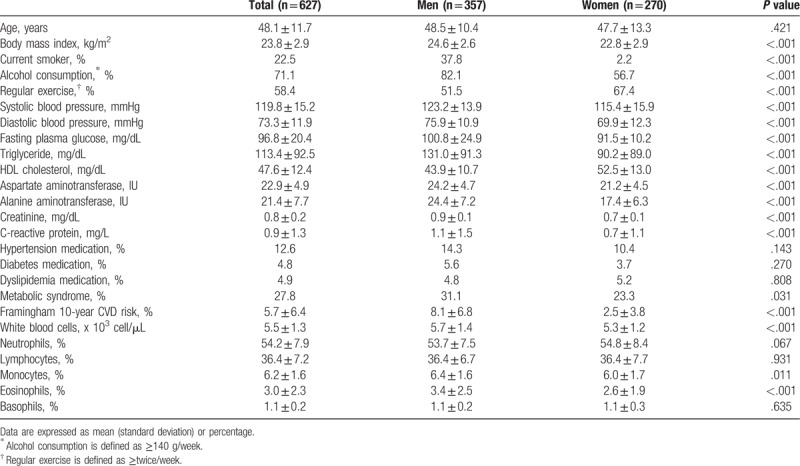
Clinical and biochemical characteristics of the study population.

Table 2 shows the correlation between Framingham 10-year CVD risk and other indicators for CVD risk. In the Pearson correlation analysis, Framingham 10-year CVD risk was correlated with age, BMI, systolic blood pressure, fasting plasma glucose, plasma triglyceride level, HDL cholesterol, monocyte count, and eosinophil count.

**Table 2:**
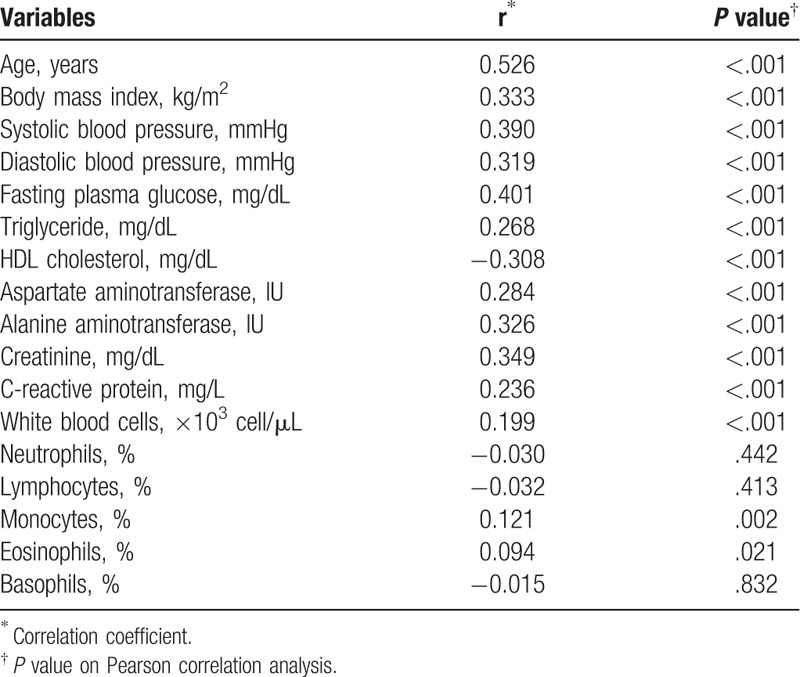
Correlation between Framingham 10-year CVD risk and indicators for cardiovascular risk.

Table 3 shows the independent contribution of WBC subtypes to Framingham 10-year CVD risk. In multiple regression model 1, the β for Framingham 10-year CVD risk was 0.266 ± 0.116 (*P* = .022) with monocyte count increment after adjusting for age, sex, BMI, and WBC count. We also assessed the association between Framingham 10-year CVD risk and monocyte count after additional adjustment for smoking status, alcohol consumption, regular exercise, systolic blood pressure, fasting plasma glucose, triglyceride, HDL cholesterol, alanine aminotransferase, creatinine, C-reactive protein, hypertension medication, diabetes medication, and dyslipidemia medication. Finally, we performed multiple logistic regression analysis in the same way after additional adjustment for metabolic syndrome. The associations were similar after using models 2, 3, and 4 (Table 3). The β for Framingham 10-year CVD risk was 0.278 ± 0.097 (*P* = .004), 0.217 ± 0.092 (*P* = .018), 0.223 ± 0.096 (*P* = .020) in models 2, 3, and 4, respectively.

**Table 3:**
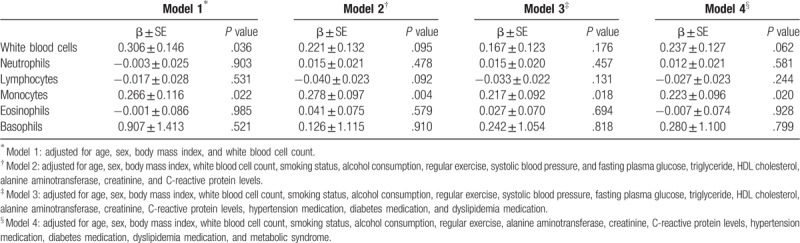
Multiple regression analysis showing the independent contribution of WBC subtypes to Framingham 10-year CVD risk.

## 4 Discussion

For community-dwelling individuals without clinical acute inflammatory disorders, our study showed a positive association of monocyte count with 10-year CVD risk estimates, independent of classical cardiovascular risk factors. These effects remained after adjusting for the presence of vascular function-modifying drugs such as anti-hypertensive drugs, lipid lowering agents, and anti-diabetic drugs.

We aimed to identify the association between components of WBC counts with CVD risk in routine clinical practice. This is the first population-based retrospective study to assess the association of all elements of WBC counts and Framingham risk score. Inflammation is a main process in the development of CVD.^[20]^ Atherosclerosis reflects the pathological substrate of CVD.^[21]^ Many mediators are involved in the regulation of interactive inflammation in atherosclerosis.^[22,23]^ Atherosclerotic plaque initiation, progression, vulnerability, and thrombosis formation are consecutive phenomena involving the interaction of lipoproteins, vascular wall components, blood cells, and the immune system,^[24]^ which are more clearly evident with blood viscosity, especially in early atherosclerosis.^[25]^ WBC count is widely used as an inexpensive, simple biomarker of systemic inflammation. It is an integrated indicator including several WBC subtypes, such as neutrophils, monocytes, lymphocytes, basophils, and eosinophils. Broadly, WBC count indicates the level of host response to stresses, the index of acute or chronic inflammatory processes.^[26]^ Therefore, WBC count has been associated with many long-term health risks, and has been shown to predict risk for all-cause mortality, cancer, and cardiovascular disease.^[27,8]^ In this study, monocyte count was associated with an increased FRS used to predict the risk of CVD over a 10-year period. Actually, a recent prospective study that examined 1000 Korean people over 6 years demonstrated that the hazard ratio for CVD mortality was 2.81 in the 3rd tertile of monocyte count compared to that in the 1st tertile.^[28]^ Also, a high monocyte count has been demonstrated to have an increased coronary artery plaque formation in subclinical stage.

The significance of our study is backed up by the fact that monocyte count is a potentially better predictor of cardiovascular disease risk than total WBC alone. Monocytes are released from the bone marrow into the blood vessels. It is a part of the mononuclear phagocyte system, with roles in inflammation and host immunity. Circulating monocytes adhere to the activated endothelium, infiltrate the vessel wall, and are exposed to growth factors, inducing their differentiation to macrophages, which ingest lipids and become foam cells.^[29]^ Atherosclerosis is initiated when the endothelium is damaged by metabolic syndrome, hypertension, diabetes, dyslipidemia, and requires subendothelial accumulation of low-density lipoprotein (LDL) cholesterol.^[30,31]^ Under metabolic stress, LDL cholesterol becomes oxidized, and oxidized LDL cholesterol worsen endothelial cell dysfunction.^[32]^ Activated endothelial cells express adhesion molecules and increase certain chemoattractants, such as interleukin-8 (IL-8) and monocyte chemoattractant protein-1 (MCP-1). This affects the recruitment of monocytes into the vascular endothelium.^[33]^ The relationship between circulating monocytes and the novel atherosclerotic plaque is significant.^[34]^ The activation of monocytes and their differentiation into lipid-laden macrophages, known as foam cells, are essential reactions in the formation of atherosclerotic lesions. Atherosclerosis is a progressive disease with a chronic asymptomatic phase characterized by inflammation and lipid accumulation in the vessel wall, leading to plaque build-up. Therefore, most people with elevated CVD risk are unaware of their critical condition.^[35]^ Macrophages, descended from monocytes, are the immune cells that regulate development, and function in host immunity and inflammation. Macrophages exist in all tissues, and phagocytize cellular debris and pathogens. Macrophages present antigens to T cells and produce cytokines to modify cells when tissues are injured. Macrophages are highly plastic and dynamic.^[36]^ The heart and blood vessels contain a number of macrophages. Macrophages capture about 8% of noncardiomyocytes in the heart. This percentage can significantly alter the underlying conditions of CVD.^[37]^ Also, metabolic syndrome, which is a combination of cardiometabolic risk factors, is generally linked to increased visceral adiposity, which can be implicated in non-alcoholic fatty liver disease and subsequently early atherosclerosis.^[38]^ Our study showed the overall prevalence of metabolic syndrome was 31.1% in men and 23.3% in women and a positive association between monocyte count and 10-year CVD risk estimates remained after additional adjusting for the presence of metabolic syndrome.

Our study has several limitations. First, since all study subjects were volunteers who had visited a single hospital for a physical evaluation, some having co-morbidity condition, the way in which the study population was enrolled may have introduced selection bias and may have failed to gather a representative sample of the general Korean population, albeit performing after excluding subjects with any acute inflammatory disease and adjusting for the presence of vascular function-modifying drugs. Second, this is a cross-sectional study without control population, so additional prospective longitudinal studies are needed to investigate the cause and effect between monocytes and the development of CVD over a longer time period.

In conclusion, taking into account the role of monocytes in atherosclerosis, our results suggest that the early detection of increased monocyte counts may be important for the assessment of cardiovascular health management. Further larger-scale prospective cohort studies are warranted to determine these associations in the future.

## Author contributions

**Conceptualization:** Jung-Hwan Kim, Yong-Jae Lee, Byoungjin Park.

**Data curation:** Jung-Hwan Kim, Yong-Jae Lee, Byoungjin Park.

**Formal analysis:** Jung-Hwan Kim, Byoungjin Park.

**Supervision:** Byoungjin Park.

**Writing – original draft:** Jung-Hwan Kim.

Byoungjin Park orcid: 0000-0003-1733-5301.
